# The meaning of autonomy when living with dementia: A Q-method investigation

**DOI:** 10.1177/1471301220973067

**Published:** 2020-12-29

**Authors:** Sarah E Wolfe, Beth Greenhill, Sarah Butchard, Jennie Day

**Affiliations:** Department of Clinical Psychology, 4591 University of Liverpool, UK; Department of Public Health and Policy, 4591 University of Liverpool, UK

**Keywords:** dementia, autonomy, rights-based approaches, Q-method, subjectivity

## Abstract

**Background and Aims:**

Sensitivity to the rights of people with dementia is a key principle cited in the World Health Organisation’s global action plan on dementia. Some critics question whether rights-based approaches embody loose and ill-defined ideas incapable of bringing about meaningful change. Exercising the right to autonomy is considered a core problem for people living with dementia. The tradition of individual sovereignty dominates ideas about autonomy, although the person as an individual is not a cross-culturally universal concept. This study explored the viewpoints of people with dementia and family carers regarding the meaning of autonomy with a view to informing rights-based practice.

**Methods:**

Twenty participants, people living with dementia and family carers, each conducted a Q-sort of statements regarding the meaning of autonomy. A by-person factor analysis was used to identify patterns in how the range of statements about autonomy were ranked.

**Results:**

Three factors emerged: retaining independence and self-expression, accepting dependence but being included and opportunity for connection. There was some agreement across these different views regarding the importance of being given time to think before making decisions and being kept active.

**Conclusions:**

This study highlights the need for a person-centred approach to supporting people with dementia to claim their rights and the importance of adopting a stance of curiosity and critical thinking in rights-based training and professional practice. The findings suggest a variety of meaningful stories of autonomy and the possibility of further developing existing rights-based frameworks for dementia care.

## Background

There are approximately 850,000 people living with dementia in the United Kingdom, with a predicted 40% increase by 2025 (Alzheimer’s Society, 2014, 2017). Autonomy is viewed as a fundamental principle underpinning human rights (Doyal & Gough, 1991), which are protected and promoted in the United Kingdom by the Human Rights Act (1998). The principles of fairness, respect, equality, dignity and autonomy (FREDA) have been offered as a human rights-based approach to healthcare that operationalises and seeks to demystify rights legislation (Curtice & Exworthy, 2010). The FREDA framework defines autonomy as ‘the principle of self-determination whereby a person is allowed to make free choices about what happens to them, that is the freedom to act and the freedom to decide, based on clear, sufficient and relevant information and opportunities to participate in the decision-making’ (Curtice & Exworthy, 2010). The loss of autonomy associated with the experience of having dementia is considered to be a core challenge for those living with the condition (DeWaal, 2014).

The importance of social context and respectful and responsive relationships for people with dementia has been highlighted (Cheston & Bender, 1999; Kitwood, 1997; Sabat & Harré, 1992). However, rights declarations have been criticised for underplaying the interdependence and interconnectedness of human experience and framing the person as a separate autonomous individual (Baldwin & Capstick, 2007). Autonomy, as referred to in healthcare and political discourse, traditionally centres on the liberal notion of self-determination and is reinforced through ideology based on independence and consumerism (Fyson & Cromby, 2013; Harding, 2012). These ideals may be at odds with the interdependent nature of care relationships when living with dementia and thus challenged through further exploration (Holstein et al., 2011).

Christman and Anderson (2005) describe the core meaning of autonomy as ‘the idea of being one’s own person, directed by considerations, desires and conditions and characteristics that...are part of what can somehow be considered one’s authentic self’ (p. 3). Kitwood’s (1997) theory of dementia recognises personhood as the human being in relation to others, being seen by others to hold a certain status and being worthy of respect. Rather than a result of maturation, personhood is thought to emerge out of interaction with and attachment to others (Agich, 2003; Holstein et al., 2011; Kitwood & Bredin, 1992). Studies exploring ‘couplehood’ and the relational self in assisted living settings support the idea that social interaction, valued social roles and supportive others contribute to the maintenance of autonomy and selfhood (Perkins et al., 2012; Wadham et al., 2015).

Human rights can be considered part of a context-dependent and socially constructed discourse (Miller, 2010), providing a framework about ‘how we might best live in a world of others’ (O’Byrne, 2012, p. 1079). According to social constructionism, traditions of meaning are ‘taken-for-granted’ assumptions that are historically and culturally situated (Gergen, 2009). The current study does not seek to label traditional western ideas of autonomy as right or wrong, rather to consider the range of views or stories that might exist about the notion of autonomy from the perspective of those living with dementia and, therefore, the relevance of commonly accepted constructions of autonomy and concepts such as relationality. The study recognises both people with a diagnosis of dementia and family carers as experts by experience who share almost all aspects of the dementia journey, including co-navigating daily living and seeking to have a voice in care planning and decision-making. Q-methodology offers a systematic means to examine human subjectivity in a manner that elicits majority and minority social discourses (McKeown & Thomas, 2013; Stainton Rogers & Stainton Rogers, 1990; Stenner et al., 2008; Watts & Stenner, 2012) and has been used successfully in previous research to elicit the views of people with dementia (Forrest, 2010; Hill et al., 2016; Westbrook et al., 2013). In this study, Q-methodology was selected to answer the following question: what does autonomy mean to people living with dementia?

## Method

### Study design and recruitment

Q-methodology consists of two stages. A variety of methods are used to generate as full a range of existing statements as possible about a topic, known as the ‘concourse’. These statements are extracted, collated and grouped into themes to produce a manageable number of representative items called a ‘Q-set’. Participants are then asked to rank the statements in a Q-set according to the extent to which they agree or disagree, otherwise known as a Q-sort, using a grid featuring a ‘forced choice distribution’. In a forced choice distribution, all participants are required to place statements in a fixed template with a pre-specified number of statements under each column of the matrix. Groups of participants who rank the statements in a similar way can then be identified using a form of ‘by-person’ factor analysis. Interpretation of these emerging factors allows for rich descriptions of the subjective viewpoints held by the participants.

Q-methodology participants are selected due to their special relevance to the goals of a study (McKeown & Thomas, 2013). Purposive sampling was used to recruit participants with experience of dementia through an older adult community team within a local National Health Service Trust. Ten participants were recruited for the interview stage including people with dementia (*n* = 4), family carers (*n* = 3) and dementia care professionals (*n* = 3). Dementia care professionals were in a position to contribute their views as part of generating the concourse to produce the Q-set but were excluded from the Q-sort stage due to the focus of the research question on the views of experts by experience. Twenty participants, including people with dementia (*n* = 11) and family carers (*n* = 9), were recruited to complete the Q-sort stage. People with dementia and carers who participated in the first stage (*n* = 7) were invited to opt in to and took part in both stages.

A briefing session was held during a team meeting to support recruitment. Short presentations were held at a service user forum and at a memory group. Most participants self-selected in response to the advertisements at these meetings. Additional participants were sought through team clinicians to increase diversity within the sample outside of these events but this did not result in wider participation.

Participants expressed their interest directly at the aforementioned research presentations or via telephone or email and were given copies of the participant information sheet to consider. The information sheet made explicit reference to the need for all participants to be able to provide consent to participate at the research appointment and how their capacity to do so might vary over time. Care coordinators were informed of expressions of interest made by people under their care who were living with dementia, which provided an opportunity for clinicians to monitor potential participants and to raise concerns regarding capacity prior to the research appointment. Capacity to participate was confirmed in advance by care coordinators and monitored at the point of participation by the researcher, who is also a clinician. At the research appointment, the participant information sheet was provided again by the researcher (S.W.). If participants agreed to take part, written informed consent was obtained at the point of participation immediately prior to completing the research tasks. If a participant was deemed to lack the capacity to consent at that point, contingency plans were in place to offer alternative means of making a contribution where appropriate, for example through providing their views verbally, and to inform their care coordinator. In practice, this contingency plan was not needed for any of the participants (Table 1).

**Table 1.** Participant characteristics.

Role	Gender	Marital status	Age range	Ethnicity	Experience of dementia	Stage
Professional	Female	Married	41–50	White British	8 years	Interview only
Professional	Female	Married	41–50	White British	22 years	Interview only
Professional	Female	Married	41–50	White British	17 years	Interview only
Person with dementia	Male	Widowed	61–70	White British	4 years	Interview and Q-sort
Daughter carer	Female	Single	51–60	White British	10 years	Interview and Q-sort
Person with dementia	Male	Divorced	71–80	White British	18 months	Interview and Q-sort
Person with dementia	Male	Married	71–80	White British	2 years	Interview and Q-sort
Spouse carer	Female	Married	61–70	White European	2 years	Interview and Q-sort
Person with dementia	Male	Married	61–70	White British	2 years	Interview and Q-sort
Spouse carer	Female	Married	61–70	White British	2 years	Interview and Q-sort
Daughter carer	Female	Married	41–50	White British	10 years	Q-sort only
Person with dementia	Female	Divorced	71–80	White British	18 months	Q-sort only
Spouse carer	Female	Married	71–80	White British	3 years	Q-sort only
Person with dementia	Female	Married	81–90	White British	3 years	Q-sort only
Spouse carer	Male	Married	81–90	White British	3 years	Q-sort only
Person with dementia	Male	Married	71–80	White British	2 years	Q-sort only
Spouse carer	Female	Married	71–80	White British	2 years	Q-sort only
Person with dementia	Male	Co-habiting	61–70	White British	3 years	Q-sort only
Person with dementia	Female	Married	61–70	White British	4 years	Q-sort only
Spouse carer	Male	Married	61–70	White British	4 years	Q-sort only
Person with dementia	Male	Married	61–70	White British	6 years	Q-sort only
Person with dementia	Male	Married	71–80	White British	10 years	Q-sort only
Spouse carer	Female	Married	61–69	White British	10 years	Q-sort only

Ethical approval for this study was granted by the NRES Committee North West and Health Research Authority on August 9th, 2016 (REC reference 16/NW/0528). This research received no specific grant from any funding agency in the public, commercial or not-for-profit sectors.

### Generating the Q-set

The Q-set was developed via a literature review and interviews involving dementia care professionals, people living with dementia and family carers. An electronic search of the literature via MEDLINE, PsycINFO, Scopus, CINAHL Plus and ScienceDirect was undertaken between October 2016 and February 2017, using the keywords ‘autonomy’ and ‘dementia’. The criteria for inclusion were articles published in peer-reviewed journals available in English, focused on any type of dementia, and featuring constructions of autonomy as expressed by participants. A selection of 35 empirical and non-empirical academic studies focussing on autonomy or closely related concepts were read in full. Statements pertaining to the meaning of autonomy were extracted and collated in a database.

An interview topic guide was developed through consultation with the research team members and was designed to explore the participants’ understanding of the term ‘human rights’, experience of dementia, roles and responsibilities, experience of decision-making and types of support. During the interview, participants were also asked for reflections in response to a quote, providing a definition of autonomy adapted from Curtice and Exworthy (2010):

‘*Autonomy is being allowed to make free choices about what happens to you, deciding this based on information and acting upon your decision’ (p. 154)*

The definition was provided as the research team felt the word autonomy might not be familiar to all participants and the use of a stimulus is not uncommon in developing the concourse in a Q‐study. This particular definition was chosen as it is widely accepted and utilised in dementia care services taking a rights-based approach. The interview guide was developed to prompt discussion about the relevance and limitations of the definition according to participants’ experiences of dementia (see Table 2).

**Table 2.** Interview topic guide.

Introductory question		
People with dementia	Carer	Professional
How long have you been living with dementia?	How long have you been supporting (*the person living with dementia*)?	How long have you been working in dementia care?
Who supports you with day-to-day activities, if anyone?	In what ways do you support them currently?	What are your main responsibilities?
What do they do to support you? (*If applicable*)	What changes have you noticed in (*the person living with dementia*) over time?	What do ‘human rights’ mean to you?
How do you find this?	What do ‘human rights’ mean to you?	
What do human rights mean to you?		
Main questions		
What does autonomy mean to you?		
‘Autonomy is being allowed to make free choices about what happens to you, deciding this based on information and acting upon your decision’. What do you understand this to mean?		
What would you expect to be able to do on this basis?		
What would you expect from others on this basis?		
How does this definition fit with your experience of living/working with dementia?		
What is the most important bit about it for you?		
What might you change or add to this definition, if anything?		
How helpful is this definition for people who are living/working with dementia?		
What other ideas do you have about what autonomy might mean to you?		
Is there anything you would like to add?		

Interviews were conducted at a local community hospital or at the participant’s home according to their preference and convenience. Couples who both wished to participate were interviewed jointly in accordance with their wishes. All interviews were audio recorded as per written consent. Interviews were transcribed verbatim by an external agency under a confidentiality agreement.

Interview transcripts were read, and statements pertaining to the meaning of autonomy were extracted. Two members of the research team (S.W. and J.D.) met to review the statements by printing out hard copies of each one and organising them into themes. Once the full range of possible themes was identified, the statements under each theme were reviewed by the team and a single sentence or phrase chosen to capture the essence of each theme. Effort was made to retain original wording and to choose statements that were clear and concise. This was a dynamic and time-consuming process which involved constant discussion between researchers and several iterations, resulting in a final Q-set of 24 statements (see Table 3).

**Table 3.** Final Q-set.

Statement
Other people taking decisions for you
Being helped to see things from different perspectives
Being free to make unwise decisions and take risks
Having a use and giving back
Being able to say no
Being given the time to think and weigh things up before making decisions
Being included in decision-making that concerns you
Needing help from professionals in order to do things
Using technology to have freedom and keep safe
Being given the chance to be listened to
Making decisions based on your values
Being given the opportunity to understand what’s happening to you
Making decisions about the small things that matter to you
Someone being with you who can make you feel good – then you can make good decisions
Having user-friendly systems
Other people knowing you and your history very well
Other people listening to what you want now
Being kept active
Being in charge of yourself, what you think and what you want
Being given resources to make free choices
Being recognised by other people as an individual with memories
Being able to express who you really are
Being able to cope with your feelings about what is happening to you
Doing the things that you did before, just with limitations

In order to ensure that the Q-set was suitable, the Q-sort procedure was piloted by a volunteer living with dementia. Subsequently, four statements were edited, and four statements were removed. The final Q-set was printed on yellow cards for better visibility, with a number representing each statement printed on the front.

### Administering the Q-sort

Q-sorts were undertaken at a local community hospital or at the participant’s home. Where couples were taking part, Q-sorts were completed individually. However, if the person with dementia wished for their partner to be present, then the carer completed their Q-sort first to reduce response bias. Observing carers were asked to refrain from interjecting while the person with dementia completed their Q-sort. The researcher supported participants with dementia by reiterating instructions and highlighting any ranking of the statements which appeared incongruous with verbal utterances.

Participants were given a sheet with the stimulus question ‘what does autonomy mean to people living with dementia?*’* and three boxes marked ‘agree’, ‘neutral’ and ‘disagree’. They were asked to read through the Q-set and form three piles according to whether they agreed, disagreed or felt neutral about each statement, drawing on their personal views and experiences. Any reflections or comments were recorded as notes.

**Figure 1.**
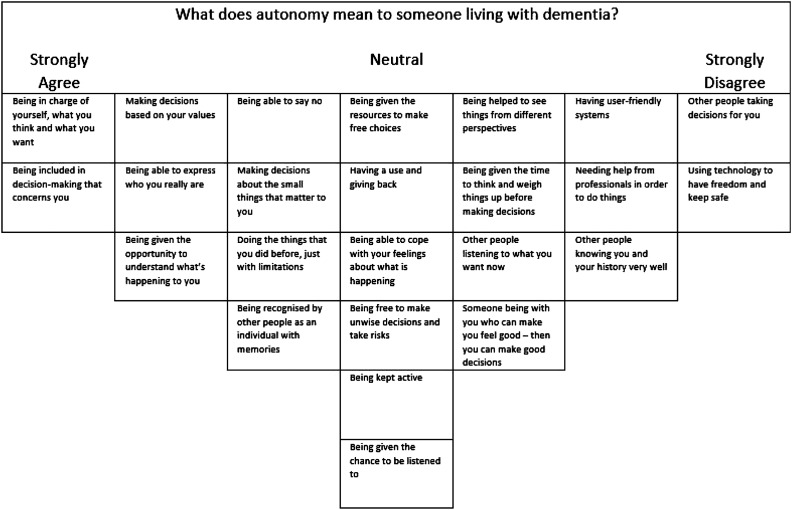
Example of a completed Q-sort grid.

The participants were then introduced to the Q-sort grid and invited to organise the statements in response to the same question indicating their level of agreement (see Figure 1). Participants started with the statements they agreed with, chose two they agreed with most strongly and placed them at the +3 position on the grid. They then placed statements across the grid along the agree continuum until all agree statements were placed. They considered the statements they disagreed with, chose two they most disagreed with most strongly and placed them at the −3 position. Finally, they placed the neutral statements around the 0 position. Participants were informed that they could move statements around until they were happy with the completed grid. Comments offered during the task were recorded as notes.

### Data analysis

In a Q study, by-person factor analysis is used to illuminate groups of people who have ranked the different statements in a Q-set in a similar way (Watts & Stenner, 2012). The covariation of the rankings made by the people within these groups is thought to indicate that their individual Q-sorts are manifestations of latent factors (Watts & Stenner, 2012). By interpreting these emergent factors using the statistical output from factor analysis alongside field notes, it is possible to understand the nature of the opinions expressed about a given topic. In the current study, by-person factor analysis was undertaken using a free software programme called PQMethod (Schmolck, 2014).

Factors were extracted using Brown’s (1980) centroid factor analysis method. An attempt was made to extract four factors in accordance with Watt and Stenner’s principle of one factor for every six Q-sorts (Watts & Stenner, 2012, p. 197). Factors with eigenvalues over 1.00 were deemed significant in line with the Kaiser–Guttman criterion (Guttman, 1954; Kaiser, 1960, 1970). However, factor loadings indicated that only three factors reached significance levels. Minimum loadings were identified via the automatic flagging feature in PQMethod.

## Results

Three factors were identified: retaining independence and self-expression, accepting dependence but being included, and opportunity for connection. The emergent factors accounted for 42% of the variance, which is ‘the proportion of the meaning and variability in a Q study that is held in common’ by the participant group (Watts & Stenner, 2012, p. 98).

The extent to which a participant’s individual Q-sort overlaps each emerging factor is represented by the factor loading, ranging between zero (no match) and one (perfect match) (see Table 4). Overall, 12 out of 20 participants loaded significantly on a factor and no participants loaded on more than one factor. Eight participants loaded on Factor 1, three loaded on Factor 2 and one participant loaded on Factor 3. Of the three who loaded on Factor 2, participant eight loaded negatively, which indicates that they expressed the opposite opinion.

**Table 4.** Factor matrix of all participants’ loadings on each factor.

Participant	Factor 1	Factor 2	Factor 3
1	.5865	−.2770	−.5437
2	.4668	−.2981	.4453
3	.5154	−.4159	.4403
4	.6967^a^	.1242	−.1723
5	.3401	−.1910	−.2731
6	.3401	.2558	.4553^a^
7	.8275^a^	−.4566	.0808
8	.2458	−.4578^a^	.3048
9	.5353^a^	.3406	.3829
10	.3942	.1441	−.1504
11	.6552^a^	−.2639	−.3289
12	.2815	−.0035	.1616
13	.1701	.5357^a^	.0494
14	.4811^a^	.1614	−.0164
15	−.1418	.3554	.2495
16	.3262	.3672	−.2657
17	.5590^a^	.1299	−.0306
18	.8907^a^	.0326	.1399
19	.2850	.4026^a^	−.1702
20	.4224^a^	.2067	−.1373
% Variance	25	9	8

^a^Significant loading determined by automatic flagging.

### Factor 1: retaining independence and self-expression

Out of the eight participants who endorsed this viewpoint, five were carers (two men and three women) and three were men living with dementia of the Alzheimer’s type. The majority were married or cohabiting and all had previously been in paid employment. There was a wider range of ages among the carers in this group than the other factors.

Self-efficacy was prominent in the responses of people who loaded significantly on this factor. The most important thing for this group was being involved in decision-making that concerned them or, ideally, exercising their own judgements and choices (see Table 5). They sought to make sense of their condition and situation and draw upon their values and sense of self-identity to manage independently day-to-day. Deciding about the small things for oneself and being able to adapt emotionally to one’s situation were also rated more positively in this factor than Factors 2 and 3.

**Table 5.** Distinguishing statements for Factor 1.

Statement	Q-sort value
Being included in decision-making that concerns you	+3
Being able to express who you really are^a^	+1
Being able to cope with your feelings about what is happening to you^a^	0
Needing help from professionals in order to do things^a^	−3

^a^Significance at *p* < .01.

This group did not express reliance upon technology and although they felt a sense of connection was important in general, they did not value being with others who could make them feel good above self-determination when it came to exercising autonomy. They were particularly averse to the over-involvement of health and social care professionals or other people taking decisions on their behalf unless necessary. This group valued person-centred care and sought the involvement of close family members when necessary.

### Factor 2: accepting dependence but being included

One female carer and one male living with vascular dementia positively loaded on this factor. A man living with Alzheimer’s loaded negatively on this factor, which means he endorsed the opposite opinion. The latter lived alone and the other two were cohabiting with their spouses.

Participants who loaded on this factor appeared to emphasise the need for support from other people, while being involved in decisions. For this group, continuing with routines was important, as well as maintaining interests and roles post-diagnosis, by working around the limitations that they confronted (see Table 6). A key aspect of this was being in the company of others day-to-day who had the skills and ability to make them feel good, particularly their life partners. The group valued user-friendly systems and willing listeners in helping them to achieve their goals but accepted that other people taking decisions on their behalf might be necessary. They preferred these people to be friends or family members whom they trusted.

**Table 6.** Distinguishing statements for Factor 2.

Statement	Q-sort value
Someone being with you who can make you feel good – then you can make good decisions^a^	+3
Doing the things that you did before just with limitations	+3
Being included in decision-making that concerns you	+2
Other people taking decisions for you^a^	+2
Having user-friendly systems	+1
Using technology and having freedom to keep safe	0
Being in charge of yourself, what you think and what you want^a^	−3
Having a use and giving back^a^	−3

^a^Significance at *p* < .01.

This group was not concerned with other people being interested in their memories and they did not consider having a use and giving back as important for autonomy. In contrast to the viewpoint expressed in Factor 1, this group did not seek to oversee themselves. They expressed neutrality about the importance of having the resources to make free choices, having access to technology and being able to take risks and they did not place as much emphasis on either being able to say no or making decisions based upon personal values, compared to people loading on the other two factors.

The participant with a negative loading on this factor expressed a strong wish to decide his own destiny. In the here and now, he associated autonomy with complete independence and making informed decisions for himself alone, which he regarded as necessary for a person living alone without support from others. The participant described difficulty with the idea of other people, including close family or partners, influencing or taking decisions for him. He described dependence on others as an inevitable but dreaded consequence of advancing dementia. The views of this participant contrasted with those expressed by participants loading on Factor 1, who preferred independence but were willing and open to seeking support from trusted family or friends as and when it was needed.

### Factor 3: opportunity for connection

The participant who loaded on this factor was an older female living with Alzheimer’s cohabiting in a deprived area. Although other participants described religious upbringings, this participant expressed a deeper commitment to her faith, which appeared to influence her attitude to her condition and her views about autonomy. She expressed a real interest in meeting new people, which she described as an opportunity for stimulation, sharing stories and being of service to others.

This person placed importance on being kept active and being supported to see things from different perspectives (see Table 7). Compared to the other two factors, this person emphasised the importance of being perceived by other people as an individual with memories, retaining the right and ability to say no and having a use and giving back. She did not seek to be included in decision-making to the same extent and preferred not to take risks. Adapting emotionally to what was happening and expressing sense of self were also not considered important.

**Table 7.** Distinguishing statements for Factor 3.

Statement	Q-sort value
Being helped to see things from different perspectives^a^	+3
Being kept active	+3
Being recognised by other people as an individual with memories	+2
Being included in decision-making that concerns you	0

^a^Significance at *p* < .01.

## Discussion

This study gave voice to experts by experience affected by dementia and in eliciting views and stories both reflecting dominant and less widely recognised discourses regarding autonomy, it has the potential to facilitate integration of new perspectives regarding autonomy as a human rights principle and social obligation (Kinderman, 2004). There has been some debate regarding whether autonomy should be conceptualised as individual or relational (e.g. Harding, 2012). However, the factors that emerged in this study challenge current influences on the language of human rights and the ways in which they are implemented in practice, namely emphasising choice and consumerism at the exclusion of a more nuanced understanding of the reality of interdependence (Fyson & Cromby, 2013). Going beyond this, the emerging factors suggest a range of perspectives indicating a more complex relationship than a purely binary individual-relational distinction.

Across the three emerging factors, there was consensus regarding the importance of being given time to weigh things up before making decisions and being kept active. The value of having adequate time to reflect and make decisions at one’s own pace has been highlighted in a recent systematic review of decision-making in persons with dementia (Wied et al., 2019). The suggestion that activity is a means through which individuals can be supported to claim their right to autonomy also echoes previous research (e.g. Harmer & Orrell, 2008).

Factor 1 emphasised independence, individuality and self-expression akin to Christman and Anderson (2005) conceptualisation of autonomy. A lack of desire for professional involvement was clear and frustrations with interference echoed real-life experiences of prescribed disengagement (Swaffer, 2016). However, the importance of having opportunities for self-expression and valued social roles was evident and is supported by theories of normal ageing and loss in dementia (Cheston & Bender, 1999; Havighurst & Albrecht, 1953). The intention to remain at the forefront of decision-making and to maintain independence despite dementia may indicate a powerful desire within some to resist the effects of the condition.

Factor 2 described adapting to life with dementia with the support of spouses and close relatives, echoing research about the role of couplehood in dementia (Perkins et al., 2012; Wadham et al., 2015) and theories of intersubjectivity (Kitwood & Bredin, 1992). Adapting to limitations and a willingness to entrust other people with decision-making may suggest greater acceptance of the realities of living with dementia and an emphasis on getting needs met wholly through relationships. In contrast, the opposite viewpoint also suggested anxiety about relying upon others to such an extent. This raises the question of whether prior experiences within relationships or attachment styles might influence views of autonomy and ways of coping. Whilst acknowledging the importance of loving and respectful relationships emphasised in other studies (Fetherstonhaugh et al., 2019), these findings raise the question of how people with dementia living alone or those with difficult attachment histories might be supported to claim their right to autonomy.

Factor 3 emphasised the importance of people taking a position of curiosity towards one another and not interacting as strangers, emphasising the importance of personal interconnections for autonomy (Kitwood, 1997; Kitwood & Bredin, 1992). It was also suggested that valuable and empowering connections might be experienced through spirituality, not just through interactions with other people, which is supported by previous research (Agli et al., 2015). These findings may provide some hopeful ideas about how to support and empower people with dementia in the absence of loved ones.

Factor 1 appears to reflect important features of the dominant discourse regarding autonomy, as found within rights-based frameworks such as FREDA (Curtice & Exworthy, 2010), in its references to inclusion, self-expression and freedom from interference by professionals. What Factor 2 adds is a story of inclusion that emphasises existing trusted and rewarding relationships as essential and preferred mechanisms for choice and action. However, a distinct and critical story also emerged strongly rejecting this notion of reliance on the ‘other’, whether relative or professional, seemingly characterised by a motivation to self-protect, greater inflexibility and higher anxiety than the more individualistic autonomy described by Factor 1. Factor 3 offers a story of connection between any two people, including non-human ‘others’, and autonomy as the fruit of their efforts to meet with mutual curiosity. Here, co-creation of opportunities to broaden thinking and enjoy pastimes is for the sake of fulfilment in the moment as opposed to achieving individual sovereignty.

### Clinical implications

The findings indicate that a ‘one-size-fits-all approach’ to thinking about and supporting matters of autonomy is of limited utility for people with dementia, as argued elsewhere (Hill et al., 2016). Assessments should take the person’s immediate social context into account and consider where action may be taken within the system of care to better support a person’s autonomy (Brown & Lloyd, 2012; Sherwin & Winsby, 2010). By taking steps to foster greater cooperation, people with dementia may be supported into a position of capability to exist and function as they wish (Nussbaum, 2006).

Staff training in person-centred behaviour and communication skills has been recommended elsewhere as valuable means of supporting decision-making (Wied et al., 2019). A recent randomised controlled trial, however, has suggested staff education regarding human rights impacts little upon quality of care and well-being of patients with dementia, although it can increase staff knowledge and improve attitudes (Kinderman et al., 2018). Whilst the current study might serve to elaborate the content of rights-based training, such training might be insufficient.

Authentic partnerships have been suggested as a means of offering better quality and relationally meaningful dementia care thus mobilising social citizenship (Dupuis et al., 2012). Greater creativity and adaptability are needed to bring this to fruition and, as this study demonstrates, to attend to and honour ‘the complex, messy, interdependent reality’ of people’s lives (Shakespeare et al., 2019). More straightforward steps might include sensitively attending to the pace of proceedings, facilitating reflection on the person’s terms, and bringing renewed appreciation to meaningful activity as a mechanism for and expression of autonomy. Opportunities and means for self-expression might involve access to certain resources or materials, a variety of activities and creativity with regards to their design and execution, with ideas coming from the community of people living with dementia. More complex changes to practice to support autonomy involve deep reflection on the understanding of and commitment to non-paternalistic cultures of care, the skills to attune to the relational ‘flavour’ of everyday social interactions within the care setting. Moving away from doing care and towards a more embodied experience of entering into the person’s social world necessitates valuing existing relationships and neither replacing nor undermining them. Actively seeking to understand the individual and the nature of their existing and most valued relationships at a more personal level with curiosity could involve greater observation of how individuals and dyads navigate their relationships and their day-to-day reality, what their interactions reveal regarding the person’s desires, needs, fears and interests. Getting to know the person and becoming fully attuned moment to moment becomes the key task of care. Connecting with and becoming known to the person in this way also requires being aware of the pull to create distance when the caring role becomes painful or challenging, demanding high reflexive skill and quality clinical supervision.

### Limitations

This study has value in helping to establish previously unheard viewpoints (Watts & Stenner, 2012). The factors that emerged in this study have been generated through responses to the statements used in the Q-set and it is possible that different factors would be extracted if other statements had been included. Additional sources could have been used to generate the concourse, including social media.

Diversity within the sample was discussed among the research team in recognition of the variety of meanings that the concept of autonomy might hold for people with different life experiences, which might be influenced by education, socioeconomic status or ethnicity. All participants were white European and most had prior employment in professional roles. Many participants were engaged in service user participation initiatives, which suggest that they were a relatively empowered and motivated group. These shortcomings may have limited the breadth of perspectives captured by the study.

Not all participants in the sample were familiar with the term autonomy at the outset of the study. The use of a definition of autonomy during the interview stage may have unduly influenced the perspectives expressed by participants. The lack of familiarity with the term autonomy is noteworthy when considering the extent to which human rights language is meaningful for people in receipt of public services (Donald et al., 2009).

### Future research

The views of neither people with learning disabilities who also have dementia nor those of people experiencing early onset dementia were explored in this study, which is a limitation and important for future investigation. Dementia care professionals were not included in the Q-sort stage. Contrasting the views of staff working within different settings and comparing perspectives of service users, carers and professionals could make a worthwhile contribution to practice. Criticism of rights-based approaches as individualistic (Baldwin & Capstick, 2007; Shakespeare et al., 2019) may have further validity where professionals assume traditional or dominant interpretations of rights language.

The findings of this study indicate that technology may not be helpful or of interest for some people with dementia, but this may reflect cohort effects and could be investigated again in future. It remains unclear what influence views of autonomy and personal values have upon how people living with dementia wish their care to be organised. Future research could seek to elicit predictors of where, how and what care people go on to receive post-diagnosis.

## Conclusions

This study elicited both dominant and less frequently heard stories about autonomy from the perspective of people living with dementia. Broadly speaking, autonomy means more than the debate between a purely independent or relational autonomy would suggest. Rights-based frameworks such as FREDA, currently used to shape practice in dementia care, need to allow for reflection on rigid assumptions behind rights terms (e.g. Department of Health, 2008). Service user preferences and the nature and role of relationships in their lives historically as well as in the present should be considered paramount when supporting people with dementia to live autonomously. Care professionals are encouraged to work collaboratively and creatively with adequate training and ongoing supervision needed to support practitioners to consider how their day-to-day experience of being with the person with dementia can maintain personhood flexibly and authentically.

## Supplemental Material

sj-pdf-1-dem-10.1177_1471301220973067 – Supplemental Material for The meaning of autonomy when living with dementia: A Q-method investigation Click here for additional data file. Supplemental Material, sj-pdf-1-dem-10.1177_1471301220973067 for The meaning of autonomy when living with dementia: A Q-method investigation by Sarah E Wolfe, Beth Greenhill, Sarah Butchard and Jennie Day in Dementia
