# Supplementary material for: The meaning of autonomy when living with dementia: A Q-method investigation
Source: Dementia (London). 2020 Dec 29;20(6):1875–90. doi: 10.1177/1471301220973067 (PMC8369913; doi:10.1177/1471301220973067)
Supplement: sj-pdf-1-dem-10.1177_1471301220973067 – Supplemental Material for The meaning of autonomy when living with dementia: A Q-method investigation [file sj-pdf-1-dem-10.1177_1471301220973067.pdf]

**Appendices**

Appendix A: Concourse literature references

Appendix B: Composite Q-sort for Factor One

Appendix C: Composite Q-sort for Factor Two

Appendix D: Composite Q-sort for Factor Three

Appendix A: Concourse Literature References

Berry, B., Apesoa-Varano, E. C., & Gomez, Y. (2015). How family members manage risk around functional decline: the autonomy management process in households facing dementia. *Social Science & Medicine, 130*, 107-114.

Birchley, G., Jones, K., Huxtable, R., Dixon, J., Kitzinger, J., & Clare, L. (2016). Dying well with reduced agency: a scoping review and thematic synthesis of the decision-making process in dementia, traumatic brain injury and frailty. *BMC Medical Ethics, 17*, 46-60.

Bollig, G., Gjengedal, E., & Rosland, J. H. (2016). Nothing to complain about? Residents' and relatives' views on a ''good life'' and ethical challenges in nursing homes. *Nursing Ethics, 23*(2), 142–153.

Burmeister, O. K. (2016). The development of assistive dementia technology that accounts for the values of those affected by its use. *Ethics and Information Technology, 18*., 185-198.

DeWaal, H. (2014). Rethinking dementia: How autonomy and control can be fostered through the development of person-centred services. *Working with Older People, 18*(2), 82-89.

Dresser, R. (2001). Advance Directives in Dementia Research. *Ethics & Human Research, 23*(1), 1-6.

Fetherstonhaugh, D,. Tarzia, L., & Nay, R. (2013). Being central to decision making means I'm still here!: the essence of decision-making for people with dementia. *Journal of Aging Studies, 27*, 143-150.

Fetherstonhaugh, D., Tarzia, L., Bauer, M., Nay, R., & Beattie, E. (2014). "The red dress or the blue?" How do staff perceive that they support decision making for people with dementia living in residential aged care facilities?. *Journal of Applied Gerontology, 35*(2), 209-226.

Gather, J. & Vollman, J. (2013). Physician-assisted suicide of patients with dementia. A medical ethical analysis with a special focus on patient autonomy. *International Journal of Law and Psychiatry, 36*, 444-453.

Glos, A. (2016). Solidarity in healthcare: the challenge of dementia. *Diametros, 49*, 1-26.

Graneheim, U., Norberg, A., & Jansson, L. (2001). An observational study focusing on a woman with dementia and `behavioural disturbances', and on her care providers. *Journal of Advanced Nursing, 36*(2), 256-265.

Haberstroh, J. (2015). Autonomy and capacity to consent in dementia. *GeroPsych, 28*(1), 5–6.

Harding, R. (2012). Legal constructions of dementia: discourses of autonomy at the margins of capacity. *Journal of Social Welfare and Family Law, 34*(4), 425-442.

Heggestad , A. K. T., Nortvedt , P., Slettebø, A. (2013). 'Like a prison without bars': Dementia and experiences of dignity. *Nursing Ethics, 20*(8), 881–892.

Hill, S. R., Mason, H., Poole, M., Vale, L., & Robinson, L. (2016). What is important at the end of life for people with dementia? The views of people with dementia and their carers. *International Journal of Geriatric Psychiatry.*

Hilton, C. & Moniz-Cook, E. (2004). Examining the personality dimensions of sociotropy and autonomy in older people with dementia: their relevance to person-centred care. *Behavioural and Cognitive Psychotherapy, 32*, 457–465.

Konig, A., Crispim-Junior, C., Gomez Urla Covella, A., Bremond, F., Derreumaux, A., Bensadoun, G., David, R., Verhey, F., Aalten, P., & Robert, P. (2015). Ecological assessment of autonomy in instrumental activities of daily living in dementia patients by the means of an automatic video monitoring system. *Frontiers in Aging Neuroscience, 7*(98), 1-11.

Mäki-Petäjä-Leinonen, A. & Juva, K. (2015). Of sound mind? Dementia and aspects of assessing legal capacity. *European Journal of Health Law*, 13-37.

Namazi, K. H. & DiNatale Johnson, B. (1992). Pertinent autonomy for residents with dementias: Modification of the physical environment to enhance independence. *The American Journal of Alzheimer's Disease and Related Disorders & Research*, 16-21.

Niemeijer, A. R., Depla, M. F. I. A., Frederiks, B. J. M., & Hertogh, C. M. P. M. (2015). The experiences of people with dementia and intellectual disabilities with surveillance technologies in residential care. *Nursing Ethics, 22*(3), 307–320.

Nys, T. R. V. (2013). The wreckage of our flesh: dementia, autonomy and personhood. In Y. Denier et al. (Eds.) *Justice, Luck & Responsibility in Health Care* (pp. 189-203). New York: Springer.

Peel, E. & Harding, R. (2015). A right to 'dying well' with dementia? Capacity, 'choice' and relationality. *Feminism & Psychology, 25*(1), 137-142.

Reamy, A. M., Kim, K., Zarit, S. H., & Whitlatch, C. J. (2011). Understanding Discrepancy in Perceptions of Values: Individuals With Mild to Moderate Dementia and Their Family Caregivers. *The Gerontologist, 51*(4), 473–483.

Ryan, T., Arnold, B. B., & Bonython, W. (). Protecting the rights of those with dementia through mandatory registration of enduring powers? A comparative analysis. *Adelaide Law Review, 36*, 355-386.

Samsi, K. & Manthorpe, J. (2013). Everyday decision-making in dementia: findings from a longitudinal interview study of people with dementia and family carers. *International Psychogeriatrics, 25*(6), 949–961.

Sanborn, B. (1988). Dementia day care: a prototype for autonomy in long term care. *The American Journal of Alzheimer's Care and Related Disorders & Research*, 23-33.

Sjöstrand, M., Eriksson, S., Juth, N., & Helgesson, G. (2013). Paternalism in the name of autonomy. *Journal of Medicine and Philosophy, 38*, 710–724.

Smebye, K. L., Kirkevold, M., & Engedal, K. (2012). How do persons with dementia participate in decision making related to health and daily care? A multi-case study. *BMC Health Services Research, 12*, 241-252.

Smebye, K. L., Kirkevold, M., & Engedal, K. (2016). Ethical dilemmas concerning autonomy when persons with dementia wish to live at home: a qualitative, hermeneutic study. *BMC Health Services Research, 16*, 21-32.

Tarzia, L., Fetherstonhaugh, D., & Bauer, M. (2012). Dementia, sexuality and consent in residential aged care facilities. *Journal of Medical Ethics, 38*, 609-613.

Van den Dungen, P., Van Kuijk, L., Van Marwijk, H., Van der Wouden, J., Van Charante, E. M., Van der Horst, H., & Van Hout, H. (2014). Preferences regarding disclosure of a diagnosis of dementia: a systematic review. *International Psychogeriatrics, 26*(10), 1603–1618.

Vogelstein, E. (2016). Autonomy and the moral authority of advance directives. *Journal of Medicine and Philosophy, 41*, 500–520.

Wilkins, J. M. (2015). More Than Capacity: Alternatives for Sexual Decision Making for Individuals With Dementia. *Gerontologist, 55*(5), 716–723.

Williams, A. (2013). Integration and independence: a new approach to autonomy and social inclusion within care home. *Mental Health and Social Inclusion, 17*(2), 70-75.

Wulff, I., Kolzsch, M., Kalinowski, S., Kopke, K., Fischer, T., Kreutz, R., & Drager, D. (2013). Perceived enactment of autonomy of nursing home residents: A German cross-sectional study. *Nursing and Health Sciences, 15*, 186-193.

Appendix B: Composite Q-sort for Factor One

| -3 | -2 | -1 | 0 | +1 | +2 | +3 |
|---|---|---|---|---|---|---|
| Other people taking decisions for you | Being helped to see things from different perspectives | Being free to make unwise decisions and take risks | Having a use and giving back | Being able to say no | Being given the time to think and weigh things up before making decisions | Being included in decision-making that concerns you |
| Needing help from professionals in order to do things | Using technology to have freedom and keep safe | Someone being with you who can make you feel good - then you can make good decisions | Making decisions about the small things that matter to you | Being given the chance to be listened to | Being given the opportunity to understand what's happening to you | Making decisions based on your values |
| | Having user-friendly systems | Other people knowing you and your history very well | Other people listening to what you want now | Being kept active | Being in charge of yourself, what you think and what you want | |
| | | Being given resources to make free choices | Being recognised by other people as an individual with memories | Being able to express who you really are | | |
| | | | Being able to cope with your feelings about what is happening to you | | | |
| | | | Doing the things that you did before, just with limitations | | | |

Appendix C: Composite Q-sort for Factor Two

| -3 | -2 | -1 | 0 | +1 | +2 | +3 |
|---|---|---|---|---|---|---|
| Having a use and giving back | Being helped to see things from different perspectives | Making decisions about the small things that matter to you | Being free to make unwise decisions and take risks | Other people listening to what you want now | Being given the time to think and weigh things up before making decisions | Someone being with you who can make you feel good - then you can make good decisions |
| Being in charge of yourself, what you think and what you want | Being able to express who you really are | Other people knowing you and your history very well | Being able to say no | Having user-friendly systems | Being included in decision-making that concerns you | Doing the things that you did before, just with limitations |
| | Being able to cope with your feelings about what is happening to you | Being recognised by other people as an individual with memories | Being given the opportunity to understand what's happening to you | Being given the chance to be listened to | Other people taking decisions for you | |
| | | Needing help from professionals in order to do things | Being given resources to make free choices | Being kept active | | |
| | | | Making decisions based on your values | | | |
| | | | Using technology to have freedom and keep safe | | | |

Appendix D: Composite Q-sort for Factor Three

| -3 | -2 | -1 | 0 | +1 | +2 | +3 |
|---|---|---|---|---|---|---|
| Being able to express who you really are | Being free to make unwise decisions and take risks | Making decisions about the small things that matter to you | Being included in decision-making that concerns you | Having a use and giving back | Being given the time to think and weigh things up before making decisions | Being helped to see things from different perspectives |
| Being able to cope with your feelings about what is happening to you | Other people taking decisions for you | Being given resources to make free choices | Being given the opportunity to understand what's happening to you | Other people listening to what you want now | Being able to say no | Being kept active |
| | Using technology to have freedom and keep safe | Having user-friendly systems | Someone being with you who can make you feel good - then you can make good decisions | Making decisions based on your values | Being recognised by other people as an individual with memories | |
| | | Needing help from professionals in order to do things | Other people knowing you and your history very well | Doing the things that you did before, just with limitations | | |
| | | | Being in charge of yourself, what you think and what you want | | | |
| | | | Being given the chance to be listened to | | | |
